# Task Offloading with LLM-Enhanced Multi-Agent Reinforcement Learning in UAV-Assisted Edge Computing

**DOI:** 10.3390/s25010175

**Published:** 2024-12-31

**Authors:** Feifan Zhu, Fei Huang, Yantao Yu, Guojin Liu, Tiancong Huang

**Affiliations:** 1 School of Microelectronics and Communication Engineering, Chongqing University, Chongqing 400044, China; 2 State Grid Chongqing Electric Power Company, Electric Power Research Institute, Chongqing 401123, China

**Keywords:** multi-agent deep learning, LLM, UAV, trajectory planning

## Abstract

Unmanned aerial vehicles (UAVs) furnished with computational servers enable user equipment (UE) to offload complex computational tasks, thereby addressing the limitations of edge computing in remote or resource-constrained environments. The application of value decomposition algorithms for UAV trajectory planning has drawn considerable research attention. However, existing value decomposition algorithms commonly encounter obstacles in effectively associating local observations with the global state of UAV clusters, which hinders their task-solving capabilities and gives rise to reduced task completion rates and prolonged convergence times. To address these challenges, this paper introduces an innovative multi-agent deep learning framework that conceptualizes multi-UAV trajectory optimization as a decentralized partially observable Markov decision process (Dec-POMDP). This framework integrates the QTRAN algorithm with a large language model (LLM) for efficient region decomposition and employs graph convolutional networks (GCNs) combined with self-attention mechanisms to adeptly manage inter-subregion relationships. The simulation results demonstrate that the proposed method significantly outperforms existing deep reinforcement learning methods, with improvements in convergence speed and task completion rate exceeding 10%. Overall, this framework significantly advances UAV trajectory optimization and enhances the performance of multi-agent systems within UAV-assisted edge computing environments.

## 1. Introduction

During recent years, unmanned aerial vehicles (UAVs) have manifested considerable practical significance in enhancing mobile edge computing (MEC) networks, effectively addressing the limitations inherent in traditional MEC systems [1].

For instance, in reference [2], the efficiency of secure computing in UAV edge systems is maximized by jointly optimizing offloading decisions and resource management. In [3], X. Liu et al. present a UAV data collection scheme that optimizes UAV trajectories using a simulated annealing algorithm to minimize redundant data collection and lower energy consumption. Reference [4] presents an inorganic-assisted ultra-reliable low-latency computation offloading framework for mission-critical IoT applications, within which the position of the drone and offloading decisions are jointly optimized. Additionally, in reference [5], the deployment of UAVs to offer computing services for terminal devices is examined, and the issue of minimizing the maximum total delay between users is addressed through optimizing the offloading ratio, user scheduling, and UAV trajectory. Still, the confined coverage area of UAVs and the non-presence of a centralized control node result in each UAV having only partial observability of the UAV-assisted MEC system. Furthermore, neither the environmental model nor its dynamics is known beforehand, which makes it difficult to formulate manageable solutions for complex environments. Furthermore, the computational complexity has a tendency to increase exponentially with the number of time slots in MEC settings, particularly in multi-UAV scenarios. Therefore, these approaches frequently demonstrate limited adaptability to complex environments.

To tackle this issue, some authors harnessed multi-agent deep reinforcement learning (MADRL) for UAV-aided MEC. MADRL optimizes offloading strategies by interacting with the environment to maximize rewards, facilitating flexible trajectory planning and adaptive task offloading for multiple UAVs without requiring expert knowledge. Value decomposition algorithms, a prominent branch of multi-agent deep learning, focus on fully cooperative, centralized training and decentralized execution (CTDE) mechanisms. These algorithms abstract the multi-UAV problem as a decentralized partially observable Markov decision process (Dec-POMDP), making them well suited for cooperative scenarios with partial observability. In [6], a value decomposition network (VDN) is proposed, which approximates the total Q value as the linear sum of the Q values obtained locally by each agent, allowing the joint action corresponding to the maximum Q value to be determined. In reference [7], the QMIX algorithm is introduced, which, compared to VDN, incorporates a constraint to ensure the monotonicity of value functions and relaxes the mapping *f* between the joint action value functions and local action value functions to some extent. However, both VDN and QMIX are limited in their applicability, as their additivity and monotonicity assumptions can only handle a small subset of decomposable MARL tasks. To address these limitations, a novel decomposition method, QTRAN, is proposed in [8], which resolves the restrictions found in both VDN and QMIX by introducing an Individual-Global-Max (IGM) condition without additional hypothetical assumptions, making it suitable for a broader range of scenarios. Furthermore, in [9], QPLEX is introduced to facilitate the learning of value functions with a linear decomposition structure by transforming IGM consistency into a straightforward value-range constraint of the dominance function. This approach allows for more specific acquisition of Q values, enabling further differentiation between whether the Q value is influenced by the state or the advantage of the action taken. Additionally, the concept of residual Q networks (RQNs) is proposed in [10], which learn to transform individual Q-value trajectories in a way that maintains the IGM criterion while offering greater robustness in decomposing action-value functions. For the UAV scenario, in [11], T. Ding et al. present an improved QMIX algorithm, UMIX, aimed at optimizing UAV trajectories and operational dynamics. S. Tan et al. [12] design a communication-assisted VDN to maximize the number and fairness of offloading tasks by optimizing UAV flight trajectories. However, both the QMIX and VDN algorithms [11,12], commonly used in UAV optimization, are constrained by the functional relationship between the partial Q value $Q_{i}$ and the total Q value $Q_{tot}$, making it challenging to effectively address the relationship between local observations and the global system state. This constraint reduces task completion rates and convergence speed in complex environments. While QTRAN [8] mitigates this limitation, its performance is hindered by numerous approximations and relaxations within the solution. Enhancing the representation networks in QTRAN can address these issues and improve overall performance.

To tackle the limitations of the QTRAN algorithm, we incorporate a large language model (LLM) to enhance the representation network and boost performance. LLMs have emerged as a ground-breaking technology, demonstrating remarkable capabilities across a broad range of natural language processing (NLP) tasks. In addition to excelling in NLP, LLMs also show immense potential for integration with large-scale training data, making them highly applicable in fields such as artificial intelligence and the Internet of Things (IoT). As an illustration, ref. [13] uses an LLM to reduce sample complexity in reinforcement learning for robotic operations. Y. Xu et al. [14] propose a multi-agent reinforcement learning model in the UAV context, utilizing an LLM to accelerate the optimization process. Additionally, Y. Han et al. [15] introduce an LLM-guided IFC deep learning reinforcement framework, leveraging an LLM as prior knowledge for DRL to speed up training and provide timely feedback to agents. Reference [16] designs an LLM-based agent structure that focuses not only on high-level conversational features but also on the reasoning and decision-making capabilities of these models. Reference [17] leverages a large LLM to acquire extensive tacit knowledge during the pre-training phase, providing valuable supplementary information; this enables the implementation of an enhanced multimodal label recommendation algorithm (LLM-MHR) based on an LLM. Wang et al. [18] employed a lightweight, open-source LLM to decompose complex navigation instructions into simple, interpretable sub-instructions and trained an RL agent to sequentially execute these sub-instructions. Leveraging these interpretable sub-instructions, the agent can more effectively learn and perform Vision-and-Language Navigation (VLN) tasks. In UAV-assisted edge computing networks, collaborative efforts among multiple UAVs are critical for efficiently accomplishing tasks and achieving operational objectives. The integration of LLMs offers a promising solution to address adaptive constraints and workload imbalances in multi-agent collaboration. By leveraging LLMs, regions are partitioned dynamically based on key factors such as UAV coverage, user distribution, task characteristics, and contextual information. This strategy optimizes network configurations, significantly improving both the task completion rate and the algorithm’s convergence speed. Additionally, inspired by recent studies [19,20], we incorporate graph convolutional networks (GCNs) with self-attention mechanisms to process information from LLM-decomposed regions. This method further strengthens the system’s representation capability and markedly improves the overall convergence speed of the algorithm.

Building upon the aforementioned background, we propose the LLM-QTRAN algorithm, which leverages LLM-based region decomposition and integrates GCNs with self-attention mechanisms to effectively address the UAV trajectory control challenge in MEC environments. The principal contributions of this work represent significant advancements in UAV-assisted edge computing, providing an efficient solution to challenges associated with trajectory optimization, task offloading, and multi-agent coordination, as detailed below.

1. Region decomposition utilizing an LLM: This paper introduces the application of LLMs in the field of UAV task assignment and trajectory optimization. By leveraging an LLM, the operational domain is divided into discrete regions, facilitating more effective and adaptive segmentation based on task characteristics and environmental variables. This decomposition enhances task offloading by providing clearer task descriptions and localization decisions, thereby representing a novel application scenario for LLMs.

2. Module collaborative design: The regional partitioning results generated by the LLM are synergistically integrated with GCNs and self-attention mechanisms. Unlike simple combinations of existing methods, this approach captures cross-regional dependencies by constructing intra-regional relationships through GCNs, thereby enhancing the global mission coordination capability of multiple unmanned aerial systems.

3. Decentralized multi-agent system architecture: By conceptualizing UAV trajectory control as a Dec-POMDP, our proposed algorithm empowers each UAV to make local decisions grounded in partial observations while ensuring coordination through LLM-guided region decomposition coupled with inter-agent communication facilitated by GCN and self-attention.

4. Practical contribution: Experimental verification demonstrates that the LLM-QTRAN algorithm significantly outperforms existing methods in terms of task completion rate and convergence speed. This indicates that the proposed design effectively addresses practical challenges and represents a substantial improvement over current approaches.

## 2. System Model and Problem Formulation

This section provides a comprehensive overview of the UAV-assisted mobile edge computing system, including a detailed discussion of its architecture and operational principles. In particular, the UAV route planning problem, which is a critical component of the system, is mathematically formulated to optimize the UAV trajectories for task offloading. The formulation incorporates various constraints and objective functions to ensure efficient task execution and system performance. For the sake of clarity and to aid in the understanding of the mathematical expressions, a summary of the key symbols and parameters used throughout this paper is presented in Table 1. This table serves as a convenient reference to quickly grasp the essential variables and their corresponding definitions.

### 2.1. Network Model

**Figure 1:**
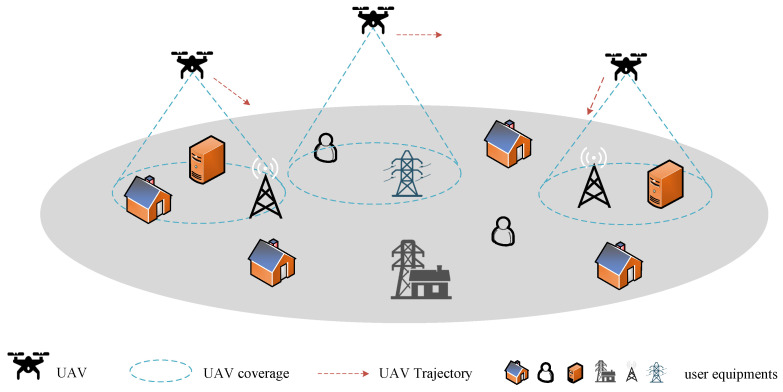
System model.

The architecture of the system is illustrated in Figure 1. In this setup, rotorcraft UAVs are utilized to offer computing services to user equipment (UE). Due to their capability to hover, take off, and land vertically, these UAVs, equipped with edge computing servers, can offload tasks with enhanced channel quality, ensuring more efficient mission execution. Let U={1,2,…,u} denote the set of UE and N={1,2,…,n} denote the set of UAVs. Due to the limited computational capabilities of IoT devices, their tasks are offloaded to UAVs. At the same time, we draw on the model design from [12,21,22], assuming that UAVs can return to rooftop charging stations for recharging before their batteries are depleted. This assumption is both reasonable and widely accepted in specific scenarios, particularly when UAVs are deployed in environments equipped with sufficient charging infrastructure. As a result, energy consumption is not a limiting factor in our model. Instead, we prioritize key performance indicators such as task completion rate and convergence speed.

The system operates in discrete time steps of duration τ. Task generation follows a predefined pattern that cannot be directly executed by the user equipment (UE). This structured approach ensures tasks are offloaded to UAVs for processing, as the UE lacks the capability to handle them efficiently on its own. At time *t*, the coordinates of the UAV *n* and the user *u* are given by $G_{n}=\left(x_{n,t},y_{n,t},z_{n,t}\right)$ and $G_{u}=\left(x_{u,t},y_{u,t},0\right)$, respectively. The Euclidean distance between the UE *u* and the UAV can be presented as $d_{u,n,t}=\sqrt{{\left(x_{n,t}-x_{u,t}\right)}^{2}+{\left(y_{n,t}-y_{u,t}\right)}^{2}+{z_{n,t}}^{2}}$.

### 2.2. Communication Model

We model the communication channel between UAVs and devices as a line-of-sight (LoS) channel. The channel gain between user *u* and UAV *n* at time slot *t* is given by

(1) $$h_{u,n,t}=d_{u,n,t}^{-\alpha }\mu ,$$

where α is assumed to be the path loss exponent, and μ is considered as the reference channel power gain at a distance of 1 m. It is assumed that all users operate over an identical bandwidth and utilize TDMA for air–ground links. The offloading data rate from user equipment *u* to UAV *n* at time *t* can be depicted as

(2) $$R_{u,n,t}=\frac{W}{\sum_{u=1}^{U}\sum_{n=1}^{N}\beta_{u,n,t}}\log_{2}\left(1+\frac{Ph_{u,n,t}}{\sigma^{2}}\right).$$

### 2.3. UAV Motion and Task Offloading Model

At time step *t*, the heading of UAV *n* is $\phi_{n,t}\in \left[0,\pi \right]$ and its normalized speed is $v_{n,t}\in \left[0,1\right]$. As a consequence, the location of UAV *n* at t+1 is

(3) $$b_{n,t+1}=b_{n,t}+\left(v_{n,t}V_{\max}+w_{n,t}\right)\tau ,$$

where $v_{n,t}=\left[v_{n,t}\cos\left(\phi_{n,t}\right),v_{n,t}\sin\left(\phi_{n,t}\right),0\right]$, and $V_{max}$ denotes the maximum allowable speed of the UAV. Additionally, $w_{n,t}$ is introduced as a random variable to account for environmental uncertainties, such as wind disturbances.

In consonance with the approach in [23,24], this paper postulates a binary computing offloading strategy. Notably, the entire computation task is compelled to be offloaded and executed comprehensively, either because of security considerations or to satisfy computational accuracy requirements [25]. Each UE is presumed to possess local tasks designated for offloading. When a UAV is within the UE’s coverage area, the UE transfers its tasks to the nearest UAV. Additionally, tasks are offloaded to the drone only if it can complete each offloaded task within the time constraint τ. Let $\beta_{u,n,t}$ indicate whether the *n*th UAV receives a task from the *u*th user at time *t*, where $\beta_{u,n,t}=1$ means the task is offloaded, and $\beta_{u,n,t}=0$ means it is not.

For the convenience of representation, the computational task of the UE is modeled as $D_{u,t}$, where $D_{u,t}\geq 0$ signifies the quantity of input data that must be offloaded.

When a UE offloads a compute task to an MEC server, the total service time comprises three key components: First, the communication time for task offloading, which is the duration required for the UE to transmit data to the MEC server; second, the computing time required by the MEC server to process the task, which depends on the server’s computational capacity and the complexity of the task; and finally, the communication time required to return the computed result, which is the duration needed to send the processed result from the MEC server back to the UE.

It is significant to note that the downstream transfer rate (i.e., the data transfer rate from the MEC server to the UE) is generally higher than the upstream transfer rate (i.e., the data transfer rate from the UE to the MEC server). This phenomenon has been empirically validated in various real-world network environments, as documented in [23].

Consequently, similar to the approaches outlined in [23,24,25], the time required to return the computed result from the edge server to the UE is typically considered negligible. This is because the high transmission rate of the downlink results in this component accounting for only a minor portion of the total service time, thereby having minimal impact on overall system performance.

The time needed to offload a task to the UAV consists of communication latency and data processing duration, as expressed by the following:

(4) $$\Lambda_{u,n,t}=\frac{D_{u,t}}{R_{u,n,t}}+\frac{D_{u,t}}{f_{u,n,t}},$$

where $f_{k,n,t}$ denotes the computational resources allocated by UAV *n* to UE *u* during time slot *t*.

### 2.4. Problem Formulation

We define a function $H\left(x\right)=\left\{\begin{array}{c} 1,\;\mathrm{if}\;x\geq 0\\ 0,\;\mathrm{if}\;x<0 \end{array}\right.$ to indicate that a UAV successfully captures a user’s task only when it is within a sufficiently close distance to the UE. Subsequently, we define the success rate of task offload as

(5) $$e_{t}=\sum_{u=1}^{U}\sum_{n=1}^{N}\left[\frac{\beta_{u,n,t}}{N}H\left(\tau -\Lambda_{u,n,t}\right)\right].$$

This paper aims to optimize the UAV trajectory to maximize the success rate of task offload, which can be mathematically expressed as

(6) $$\max_{\phi_{n,t},\nu_{n,t}}E\left[\sum_{t=1}^{T}\left(e_{t}\right)\right].$$

(6a) $$\beta_{u,n,t}\left|\right|G_{n,t}-G_{u,t}{\left|\right|}^{2}\leq r_{\max}^{2}+H^{2},\forall u\in U,t\in T,$$

(6b) $$∥G_{n,t}-G_{n,t-1}∥\leq d_{\max},\forall t\in T,$$

(6c) $$\sum_{u=1}^{U}\beta_{u,n,t}f_{u,n,t}\leq f_{n}^{\max},\forall t\in T,$$

(6d) $$\sum_{u=1}^{U}\beta_{u,n,t}\leq N_{n}^{\max},\forall t\in T,$$

(6e) $$\beta_{u,n,t}\in \left\{0,1\right\},f_{a,u,t}\in \left[0,f_{n}^{\max}\right],$$

Constraint (6a) ensures that UE associated with UAVs remains within the UAVs’ coverage area, which is defined by $r_{\max}$. Constraint (6b) constrains the flight distance of UAVs in each time slot to be within its maximum range, $d_{\max}$. Constraint (6c) restricts the computational load of UAVs to their maximum processing capacity. Constraint (6d) imposes a limit on the maximum amount of UE that each UAV can serve. Finally, constraint (6e) determines the feasible boundaries for the optimization variables. The problem presented in Equation (6) is a mixed-integer non-convex one with multiple optimization variables, making it challenging to solve by traditional optimization approaches. Fortunately, LLM-enhanced MARL is capable of exploring a vast policy space to discover the optimal solution.

## 3. LLM-QTRAN Algorithm

This section begins by outlining the MARL setup, detailing the environment, agents, state spaces, action spaces, and reward structure utilized within the UAV-assisted edge computing system. Following this, we introduce the proposed LLM-QTRAN algorithm, explaining its mechanisms, including the integration of the LLM for region decomposition and the role of the QTRAN framework in addressing challenges in decentralized control. Additionally, we provide an in-depth analysis of the network structure, encompassing the use of the GCN and self-attention mechanisms to process region-specific data and enhance the overall performance of the algorithm.

### 3.1. Task Offloading with MARL

In this paper, the fully cooperative multi-agent task is conceptualized as a DEC-POMDP, in accordance with previous studies [6,7,8]. The DEC-POMDP is expressed as G=<S,A,P,r,Z,O,N,γ>. Here, s∈S represents the state of the environment. Agent n∈N selects an action $a_{n,t}\in A$ at each time step, forming a joint action vector $a:={\left[a_{i}\right]}_{i=1}^{N}\in A^{N}$. The function $P\left(s^{\prime }|s,a\right):S\times A^{N}\times S\mapsto \left[0,1\right]$ governs all state transitions. Every agent possesses the same joint reward function $r\left(s,a\right)$, γ∈[0,1) is the discount factor, each agent generates local observations based on the observation function $O\left(s,n\right)$, and each agent also maintains an observation history δ to constrain the policy.

Training and execution: The most straightforward manner for training in MARL tasks is independent Q-learning, through which each agent acquires its action-value function independently. Despite the simplicity and scalability of this method, it lacks guaranteed convergence, even with an infinite number of exploration steps. CTDE enables agents to learn and construct individual action-value functions, ensuring that the collective optimization of each agent’s actions enhances the joint action-value function. Consequently, agents can select the optimal actions during execution by referring only to their own action-value function, without the necessity to access federated information. Even under conditions of partial observability and limited communication among agents, essential information remains accessible to all agents during training.

Next, we provide a comprehensive description of the observation, action, and reward settings in the proposed framework, outlining how each component is designed to enable efficient decision making and optimize UAV trajectory planning in the MEC environment.

Observation: The offloading strategy and data rate are contingent upon the distance between the UAV and the user; accordingly, the observation vector comprises the coordinates of both the UAV and the user. The observation vector for UAV *n* can be depicted as

(7) $$O_{n,t}=\left[\begin{array}{c} d_{1,t}^{ue},\ldots ,d_{u,t}^{ue},d_{1,t}^{uev},\ldots ,d_{n,t}^{uev},pos_{1},\ldots ,pos_{b},V_{n} \end{array}\right],$$

where $d_{u,t}^{ue}$ represents the distances between agent *n* and user, and $d_{n,t}^{uav}$ represents the distances between UAV *n* and the other UAVs. $pos_{b}$ denotes the position of obstacles, and $V_{n}$ represents the velocity of UAV *n*.

Action: Each UAV agent’s actions include its heading and speed, i.e., $a_{n,t}=\left[\phi_{n,t},v_{n,t}\right]$.

Reward: Considering the cooperative nature of the tasks, the team reward is allocated among all agents. The reward function is defined as follows:

(8) $$r_{n,t}=\alpha e_{t}-\beta d_{n,u^{\prime }}^{\min}-\sum_{n=1}^{N}\frac{c_{n}^{\mathrm{col}}}{N},$$

where $c_{n}^{\mathrm{col}}$ is the penalty term to avoid collisions, and $d_{n,u^{\prime }}^{\min}$ ensures that the agent can complete the task with a short path.

### 3.2. LLM-QTRAN Algorithm and Network Architecture

The following illustrates the design of the agent network and the LLM-QTRAN algorithm.

This paper examines a category of sequential decision-making tasks that are capable of being decomposed during the intense training phase. We initiate from [8], which elaborates on the Individual-Global-Maximum (IGM) principle.

**Definition 1.**  *Regarding a total action-value function $Q_{\mathrm{tot}}:\Delta^{N}\times A^{N}\to R$, where $\delta \in \Delta^{N}$ constitutes a total action observation history, if there exist individual action-value functions ${\left[Q_{i}:\Delta \times A\to R\right]}_{i=1}^{N}$ such that the following holds:*

(9) $$\arg\max_{a}Q_{\mathrm{tot}}\left(\delta ,a\right)=\left(\begin{array}{c} \arg\max_{u_{1}}Q_{1}\left(\delta_{1},a_{1}\right)\\ \vdots \\ \arg\max_{u_{N}}Q_{N}\left(\delta_{n},a_{N}\right) \end{array}\right),$$

then, we contend that $Q_{i}$ satisfies IGM for $Q_{\mathrm{tot}}$ under δ. In this case, $Q_{tot}\left(\delta ,a\right)$ is also asserted to be decomposable by $\left[Q_{i}\left(\delta_{i},a_{i}\right)\right]$. Fundamentally, an optimal joint action among agents is equivalent to a collection of individually optimal actions for each agent. If $Q_{tot}\left(\delta ,a\right)$ in a specific task exhibits decomposability for all $\delta \in \Delta^{n}$, the task is deemed decomposable.

For the given observation δ, take into account an arbitrary factorizable $Q_{tot}$. To effectively forge a connection between local observations and the global state while meeting the IGM condition, we adopt the approach from [8] to define $Q_{tot}$, $Q_{alt}$, $Q_{i}$ and the compensation term *V*.

(10) $$\sum_{i=1}^{N}Q_{i}\left(\delta_{i},a_{i}\right)-Q_{tot}\left(\delta ,a\right)+V\left(\delta \right)=\left\{\begin{array}{cc} 0 & a=\bar{a},\\ \geq 0 & a\neq \bar{a}, \end{array}\right.$$

(11) $$V\left(\delta \right)=\max_{a}Q_{tot}\left(\delta ,a\right)-\sum_{i=1}^{N}Q_{i}\left(\delta_{i},a_{i}\right)$$

(12) $$Q_{alt}\left(\delta ,a\right):=\sum_{i=1}^{N}Q_{i}\left(\delta_{i},a_{i}\right).$$

Here, $\bar{a}$ constitutes the set of locally optimal actions $a_{i}$. The decomposition approach is structured based on the additive composition of $Q_{alt}$ from $\left[Q_{i}\right]$, where $\left[Q_{i}\right]$ conforms to the IGM property of the new joint action-value function $Q_{tot}^{0}$. This implies that $\left[Q_{i}\right]$ acts as the individual action-value function in the decomposition of $Q_{alt}$. Given $\arg\max_{a}Q_{tot}\left(\delta ,a\right)=\arg\max_{a}Q_{alt}\left(\delta ,a\right)$, the $\left[Q_{i}\right]$ terms satisfying Equation (11) provide the precise factorization of $Q_{alt}\left(\delta ,a\right)$. Furthermore, V(δ) denotes the compensation for the discrepancy between the local observation and the global state, while $Q_{alt}$ serves as an auxiliary function to substitute for the global value function.

An interpretation of this approach lies in the fact that instead of directly factorizing $Q_{tot}$, we introduce an alternative joint action-value function, $Q_{alt}$, which is factorized through additive decomposition. Here, V(δ) compensates for the disparity between the centralized joint action-value function $Q_{tot}$ and the sum of individual action-value functions $\left[Q_{i}\right]$. This disparity is brought about by the agents’ partial observability. With full observability, *V* could be set to zero while still preserving the validity of the definitions.

The QTRAN algorithm utilized in UAV optimization effectively mitigates the constraints between local observations and the global system state. Nevertheless, its performance is constrained by various approximations and relaxations inherent in its solutions. Enhancing the representation network within QTRAN addresses these shortcomings, resulting in an overall improvement in system performance.

**Figure 2:**
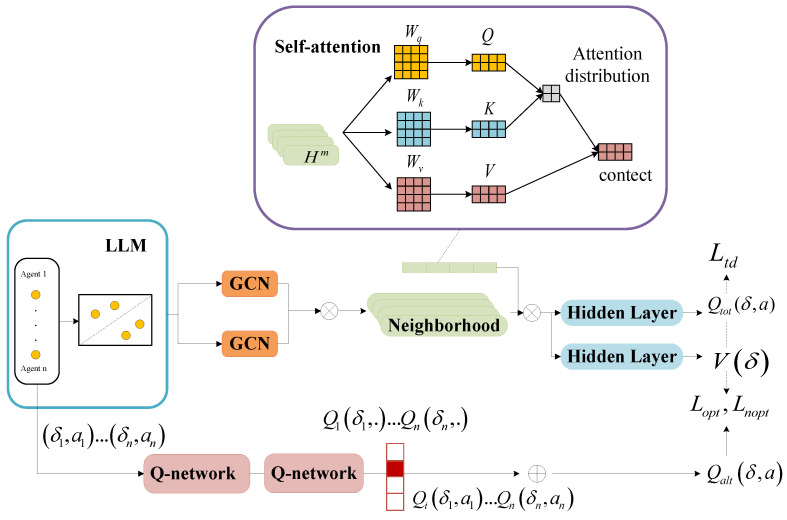
Network architecture of the LLM-QTRAN algorithm.

We enhance the QTRAN execution network by introducing three modules, as in Figure 2: LLM, GCN, and self-attention mechanism.

The LLM module within our framework is meticulously designed to facilitate macro-level decision making by analyzing task characteristics and evaluating the current system state. Specifically, the UAV swarm is prepartitioned into *n* distinct subsystems, a configuration that capitalizes on the LLM’s proficiency in high-level spatial analysis and regional partitioning.

In addition, the LLM module integrated into our framework represents a sophisticated pre-trained model, thereby obviating the necessity for supplementary training. By harnessing its extensive repository of pre-existing knowledge, the LLM furnishes task-specific priors that facilitate high-level decision making, particularly in contexts demanding swift adaptation. In this regard, the role of the LLM is not to learn from inception but to leverage its pre-trained capabilities for informed macro-level decisions. This empowers it to delineate regions, anticipate task requirements, and optimize UAV deployment without incurring the computational overhead typically associated with model training. As a result, the LLM functions as an advanced cognitive layer that steers strategic operations while enhancing system responsiveness and overall efficiency. This methodology not only optimizes the resource allocation but also bolsters the responsiveness of the UAV-assisted edge computing network.

The LLM segments regions by considering task characteristics and environmental variables to optimize task execution and resource allocation. In this process, the LLM formulates a partitioning strategy through the analysis of inputs such as computational requirements, user distribution, drone coverage, and resource capabilities. Key parameters encompass time constraints, data volume distribution, computational demands, current drone location, coverage area, and resource utilization.

The LLM leverages these inputs to dynamically adjust the zoning scheme, ensuring efficient utilization of UAV resources while accommodating mission and environmental constraints. This adaptability enhances scalability and performance across diverse dynamic scenarios. The zoning process adheres to three primary principles:

1. Maximizing task completion rate to ensure timely execution of tasks.
2. Minimizing communication overhead to reduce interactions between drones.
3. Achieving cross-regional load balancing to prevent resource congestion.

The output of the LLM is a customized area division policy tailored to task features, with area division conducted in accordance with specified principles.

After the LLM completes the region division of the drone cluster, the GCNs capture spatial dependencies and characterize each region by generating multiple neighborhoods. This step is crucial for modeling the interactions of drones both within and across regions.

GCNs excel in identifying spatial correlations between task nodes, providing a natural advantage in capturing internal relationships within the model region. Specifically, GCNs effectively capture local dependencies between nodes by aggregating information from neighboring nodes to update the feature representation of each node. This characteristic makes GCNs particularly adept at task assignment and trajectory planning in complex network structures.

The process involves several key steps: state extraction (gathering relevant state information), consolidation, correlation graph generation, and message passing. During the correlation graph generation phase, the neural network is trained to generate graphs that represent the relationships between drones and between drones and environmental objects. The absolute weight of the edges in these graphs indicates the importance of the interactions: higher edge weights between drones (e.g., between drone A and drone B or between drones and environmental objects) signify greater contributions to the successful completion of drone A’s mission.

This graphical information is then passed to the decision component, which leverages these relational data to guide task execution. By integrating these detailed representations of drone interactions, the system can make more informed decisions, thereby enhancing the overall efficiency and effectiveness of task execution.

Let $H^{0}$ represent the initial feature vector that encompasses all regions. These regional features are then processed by the GCN, which iteratively aggregates and transforms the information to derive enriched neighborhood features. This iterative process enables the propagation of spatial information across adjacent regions, effectively capturing the interdependencies between UAVs and the environment. This can be expressed using the following recursive formula:

(13) Hl+1=ReLUD−1/2AD−1/2HlWl,

where D is the degree matrix, A is the normalized adjacency matrix, $W^{l}$ is the learnable weight matrix of the lth layer, and the final output neighborhood feature is denoted as H.

Subsequently, a self-attention mechanism is then integrated into the generated *m* neighborhoods, enabling the model to dynamically assign varying degrees of importance to each neighborhood based on its relevance to the overall task.

Self-attention mechanisms excel at modeling cross-regional dependencies and can effectively handle interactions between remote tasks. Unlike traditional neighbor-based methods, which rely on fixed spatial proximity, the self-attention mechanism dynamically measures the strength of associations between different nodes, regardless of their spatial adjacency. This capability allows the self-attention mechanism to capture interactions at a broader scale, particularly in scenarios where global information needs to be considered.

This mechanism enhances the ability of unmanned aerial systems to capture subtle dependencies within the network, which is crucial for accurately accomplishing missions. By allowing the model to assess the relative importance of each neighborhood in real time, the self-attention mechanism dynamically adjusts its focus to those neighborhoods that have the most significant impact on the task at hand. Consequently, this leads to a more refined and adaptable decision-making process, improving both the precision and efficiency of task execution.

By computing weighted relationships between neighborhoods, the self-attention mechanism ensures a more precise representation of the global context, taking into account not only the local neighborhood features but also the broader interdependencies across the UAV network. This attention-based approach improves the system’s adaptability to varying environmental conditions and task requirements, ensuring that key neighborhoods exert an appropriate level of influence during the decision-making process. The weighted relationships computed by the self-attention mechanism allow the UAV system to prioritize critical interactions, ultimately leading to better performance in task allocation and execution.

Weight distribution constitutes a fundamental component of the attention mechanism. By judiciously assigning weights, the model can concentrate more effectively on the most salient aspects of the input data, thereby enhancing overall performance. In a multi-UAV system, individual UAVs may undertake distinct tasks or roles, with the observational data from certain UAVs being pivotal to the decision-making process of the entire system. By incorporating the attention mechanism, we can allocate higher weights to these critical data, enabling the model to prioritize these essential inputs during information processing, and consequently improving the accuracy and efficiency of task execution.

The weight calculation formula for this process is expressed as follows:

(14) $$H_{w}^{m}=Att\left(Q,K,V\right)=softmax\left(\frac{{\mathrm{QK}}^{⊤}}{\sqrt{a}}\right)V$$

(14a) $$Q=H^{m}W_{q}$$

(14b) $$K=H^{m}W_{k}$$

(14c) $$V=H^{m}W_{v},$$

where $W_{q}$, $W_{k}$, and $W_{v}$ are the learnable weight matrices, and a is the scaling factor of attention.

Collectively, these three modules work in tandem to improve task completion rates, reduce communication overhead, and enhance the overall performance of the UAV-assisted edge computing system.

The intensive training phase encompasses two principal aims. The first aim is to train the total action-value function, $Q_{tot}$, to precisely assess the true action-value. The second aim is to ensure that the transformed action-value function, $Q_{alt}$, perpetually conforms to $Q_{tot}$, thereby guaranteeing that the optimal actions remain unchanged. To achieve this, we formulate a global loss function within LLM-QTRAN, integrating three individual loss functions with corresponding weightings:

(15) $$L\left(\delta ,a,r,\delta^{\prime },\theta \right)=\left(L_{\mathrm{td}}+\lambda_{\mathrm{opt}}L_{\mathrm{opt}}+\lambda_{\mathrm{nopt}}L_{\mathrm{nopt}}\right)H_{w}$$

(16) $$L_{\mathrm{td}}\left(;\theta \right)={\left(y^{\mathrm{dqn}}\left(r,\delta^{\prime };\theta^{\prime }\right)-Q_{tot}\left(\delta ,a\right)\right)}^{2}$$

(17) $$L_{\mathrm{opt}}\left(;\theta \right)={\left(Q_{alt}\left(\delta ,\bar{a}\right)-{\hat{Q}}_{tot}\left(\delta ,\bar{u}\right)+V_{\mathrm{jt}}\left(\delta \right)\right)}^{2}$$

(18) $$L_{\mathrm{nopt}}\left(;\theta \right)={\left(\min\left[Q_{alt}\left(\delta ,a\right)-{\hat{Q}}_{tot}\left(\delta ,a\right)+V_{\mathrm{jt}}\left(\delta \right),0\right]\right)}^{2}.$$

Here, *r* represents the reward for action *a* during the transition from the observed history τ to $\tau^{\prime }$. The target network parameter θ employs the same structure as the agent network but possesses distinct parameter magnitudes. $L_{\mathrm{td}}$ mitigates the TD error while learning $Q_{tot}$. $L_{\mathrm{opt}}$ and $L_{\mathrm{nopt}}$ denote the losses based on whether the decomposed total Q value meets the system requirements. $L_{\mathrm{opt}}$ verifies if the selected action in the sample satisfies $a=\bar{a}$ and $L_{\mathrm{nopt}}$ checks whether the action satisfies $a\neq \bar{a}$ in Equation (10). The procedure is elaborated on in Algorithm 1.
**Algorithm 1** The LLM-QTRAN Algorithm1:Initialize replay memory *D*2:Initialize $Q_{i}$, $Q_{tot}$ and *V* with random parameter θ3:Initialize target parameters θ = $\theta^{\prime }$4:**for** episode 1 to max-episode-number **do**5:   Reset the environment, and obtain the initial local observation $O_{n}$ for each agent6:   **for** *t* = 1 to *T* **do**7:       Use LLM to region decompose the system into *K* subsystems based on observation $O_{n}$8:       Feeding *K* into GCN and iterating *l* times using Equation (13)9:       Obtain the weight values of each neighborhood by using Equation (14)10:      Select a random action $a_{n}$ for each agent with probablity ε11:      Otherwise $a_{n,t}=\arg\max Q_{i}\left(\delta_{n,t},a_{n,t}\right)$ for each agent12:      Take action $a_{n,t}$ and retrieve next observation $o_{t+1}$ and $r_{t}$ reward13:      Store transition $\left(\delta_{t},a_{t},r_{t},\delta_{t+1}\right)$ in *D*14:      Sample a random minibatch of transitions $\left(\delta_{t},a_{t},r_{t},\delta_{t+1}\right)$ in *D*15:      Set $y^{\mathrm{dqn}}\left(r,\delta^{\prime };\theta^{\prime }\right)=r+\gamma Q_{tot}\left(\delta^{\prime },{\bar{a}}^{\prime };\theta^{\prime }\right)$, ${\bar{a}}^{\prime }={\left[\arg\max_{\alpha_{i}}Q_{i}\left(\delta_{i}^{\prime },a_{i};\theta^{\prime }\right)\right]}_{i=1}^{N}$16:      Update θ by minimizing the loss Equation (15)17:      Update target network parameters $\theta^{\prime }$ with period *t*18:   **end for**19:**end for**

## 4. Simulation Results

This section presents a series of simulations designed to evaluate the performance of the LLM-QTRAN algorithm against three benchmark algorithms. A controlled variable experimental design, as described in [7], was employed to assess the validity and effectiveness of the proposed method. The QTRAN algorithm [8] underwent ablation studies and was compared with the fine-tuned QMIX [26] (an optimized version of QMIX) and K-means QTRAN. The experiments systematically varied the number of UAVs and users involved to comprehensively verify the effectiveness of the proposed algorithm.

The primary performance metrics utilized in these experiments include cumulative rewards and task offloading success rates. The reward metric quantifies the cumulative rewards accrued by agents over time, reflecting their effectiveness in achieving desired objectives. The task offloading success rate assesses the proportion of tasks successfully completed through efficient delegation and resource allocation. These two indicators are critical for evaluating the practicality and efficiency of the LLM-QTRAN algorithm in real-world scenarios. The selection of these performance metrics is informed by the methodology outlined in [7,27].

### 4.1. Simulation Parameter Setting

To evaluate the performance of the proposed scheme, we conduct a simulation verification using Python 3.8.0 within the Multi-Agent Particle Environment (MPE) framework. The simulations are performed on a Windows platform. We evaluate a 500×500 $m^{2}$ area where UAVs can detect users and offload their tasks successfully only if the horizontal distance to the UAVs is less than $r_{\max}=50\;m$; each UAV can serve a maximum of two users. For the channel model, the parameters are set to α=2 and μ=−3 dB, following the specifications in [12,28]. The noise power is set at $\sigma^{2}=-90$ dBm, with transmission power P=100 mW, and the UAVs’ maximum speed is limited to $V_{\max}=30$ m/s, as in [29]. The UAV movement is discretized with directions {0,π/4,…,7π/4} and speed ratios {0,0.5,1}. The LLM used in the model is OpenAI’s open-source large language model DeepSeek. We divide the system into two subsystems based on the UAV characteristics to manage task allocation effectively. The learning rate is set to 0.005, with a discount factor γ of 0.99. Each experimental run is trained over 1,000,000 time steps to ensure robust performance assessment. For the sake of clarity, a summary of the key symbols utilized is presented in Table 2. The parameter simulation in the table is based on [24,28,29].

In the simulation experiments conducted for this study, the configuration parameters for all algorithms were established in accordance with the specifications detailed in the methodology section. To ensure the robustness and reliability of the experimental results, extensive simulations were carried out under a range of conditions, including varying numbers of UAVs and users.

### 4.2. Benchmark Algorithms

To appraise and demonstrate the effectiveness of the LLM-QTRAN algorithm, this section considers three benchmark algorithms for comparison.

Fine-tuned QMIX: The fine-tuned QMIX algorithm enhances the classic QMIX for multi-agent cooperative tasks through key code-level optimizations and a reevaluation of the monotonicity constraint. By adjusting optimizers, replay buffer size, and exploration parameters, fine-tuned QMIX achieves better stability and convergence in complex environments. In studies related to deep reinforcement learning, this algorithm is frequently used as a benchmark for comparison [28].

QTRAN: QTRAN is an advanced factorization approach in MARL, which is designed to overcome the limitations of previous algorithms such as VDN and QMIX that rely on restrictive additivity and monotonicity constraints. QTRAN transforms the joint action-value function into a new form, enabling a more flexible factorization without these constraints. This transformation allows QTRAN to optimize tasks that are not easily factorizable under traditional methods, thus broadening the applicability of MARL in complex environments [8].

K-means QTRAN: K-means QTRAN represents an optimization of the traditional QTRAN algorithm by introducing a k-value based on the distances between UAVs, which allows the formation of k distinct clusters. Compared to the conventional QTRAN algorithm, this approach enhances the network’s representation capacity.

### 4.3. Simulation Results

**Figure 3:**
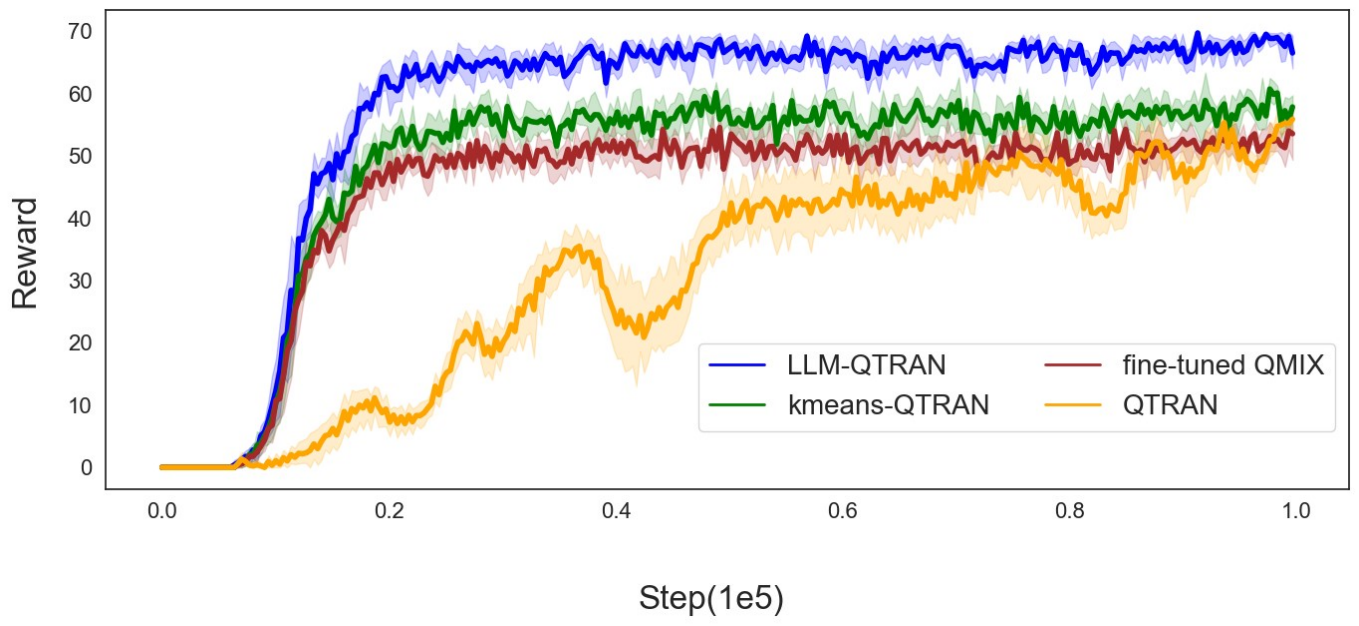
Average reward of the LLM-QTRAN algorithm and other baselines.

In Figure 3, we present a comparative analysis of the average rewards attained by the various methodologies. It is evident that both LLM-QTRAN and K-means QTRAN outperform the fine-tuned QMIX and standard QTRAN algorithms. This performance enhancement can be attributed to several factors, including a more robust network representation, effective regional decomposition of the system, and more efficient task offloading, all contributing to higher overall rewards. Specifically, LLM-QTRAN achieves the highest average reward due to its integration of pre-trained LLM parameters that encapsulate prior knowledge across diverse tasks.

Compared with the K-means QTRAN algorithm, the LLM-QTRAN algorithm leverages the strengths of LLM to dynamically determine the optimal value of *k* for clustering based on task-specific contexts and natural language features. By incorporating this advanced partitioning approach, LLM-QTRAN achieves higher rewards, outperforming conventional methods in UAV-assisted edge computing applications.

In contrast, QTRAN’s performance lags behind that of fine-tuned QMIX due to its reliance on significant relaxation and approximation techniques, which constrain its effectiveness compared to the more refined approach employed by the fine-tuned QMIX algorithm.

At the same time, it can also be seen from Figure 3 that LLM-QTRAN not only achieves higher rewards but also demonstrates a faster convergence rate. This enhanced performance can be attributed to the task priors provided by the LLM, which allow the agent to learn more efficiently and converge more quickly. By leveraging this prior knowledge, the agent is able to make more informed decisions, accelerating the learning process and facilitating the attainment of the maximum reward.

**Figure 4:**
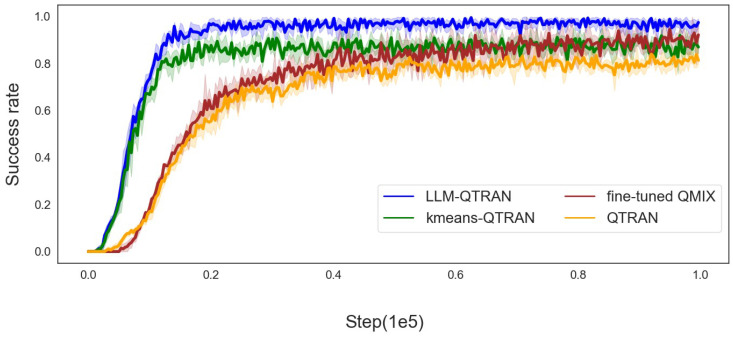
Success rate for 5 users with 2 UAVs.

**Figure 5:**
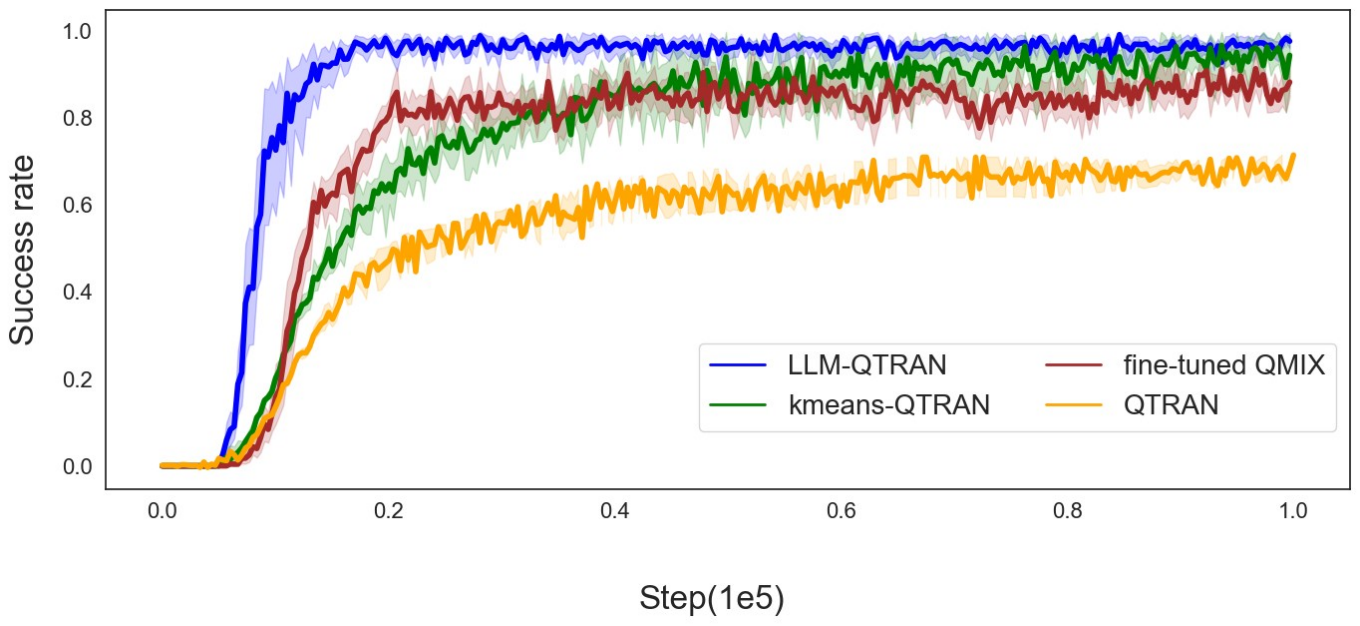
Success rate for 7 users with 3 UAVs.

**Figure 6:**
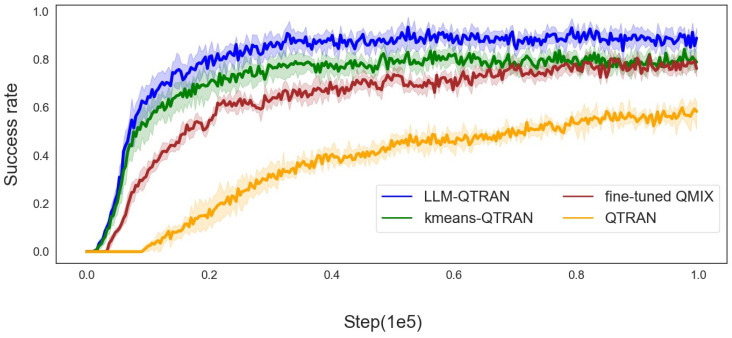
Success rate for 10 users with 4 UAVs.

Figure 4, Figure 5 and Figure 6 further demonstrate the mission offloading success rates of the LLM-QTRAN algorithm, in comparison to other benchmark methods, across various configurations of UAVs and users. The results clearly highlight that LLM-QTRAN exhibits superior stability and adaptability in fluctuating operational environments, consistently outperforming the QTRAN algorithm and the fine-tuned QMIX algorithm. This enhanced performance can be attributed to the advanced network representation capabilities embedded within the LLM-QTRAN framework, which enable it to efficiently adapt to a wide range of operational scenarios and environmental changes.

Notably, LLM-QTRAN consistently achieved the highest task completion rate across all experimental settings. This remarkable performance can be attributed to the integration of pre-trained, task-specific knowledge in the LLM, which enhances the precision of task classification and region decomposition. Compared with the K-means QTRAN algorithm, which requires manually predefining the *K* value and considering the distance between each UAV for decision making, LLM-QTRAN optimizes task unloading decisions with higher accuracy by leveraging contextual information and specific task characteristics. Consequently, LLMs are capable of autonomously managing complex decision-making processes, significantly reducing reliance on human input or predefined parameters, and further improving task offloading efficiency.

In Table 3, we present the key performance indicators of the comparison algorithms illustrated in Figure 3, Figure 4, Figure 5 and Figure 6, including task completion rate and convergence speed. These metrics have been averaged to provide a clearer and more comprehensive overview of the results.

Overall, the experimental results clearly demonstrate that the LLM-QTRAN algorithm substantially improves mission unloading success rates and outperforms baseline algorithms. These findings provide strong evidence of its superior performance in UAS-assisted edge computing systems, highlighting LLM-QTRAN’s significant potential for mission-critical offloading, especially in dynamic and resource-constrained environments.

## 5. Conclusions

UAVs equipped with computing servers offer an effective solution for ground users to offload computationally intensive tasks, particularly advantageous in remote or resource-constrained environments where conventional edge computing resources may be scarce. However, most existing methodologies encounter limitations due to their dependence on centralized controllers, which introduce significant communication overhead, or fail to adequately align local observations with the global state of UAV clusters. This misalignment frequently diminishes task-solving efficiency, leading to suboptimal task completion rates and slower convergence.

To tackle these challenges, this study introduces a novel multi-agent deep learning framework aimed at optimizing multi-UAV trajectory planning within a Dec-POMDP. By integrating the QTRAN algorithm with an LLM for intelligent region decomposition, the framework employs a GCN alongside self-attention mechanisms to effectively capture and manage inter-subregion relationships, thereby enhancing the network’s representational capabilities.

The simulation results demonstrate that the proposed framework significantly outperforms traditional MADRL methods in terms of convergence speed and task completion rate. Specifically, compared to fine-tuned QMIX and K-means QTRAN, LLM-QTRAN achieves 18.2% and 28.0% faster convergence, respectively, along with 16.7% and 12.4% higher task success rates. Relative to QTRAN, LLM-QTRAN exhibits a 66.7% improvement in convergence speed and a 32.9% increase in task success rate. These findings highlight the framework’s effectiveness in UAV trajectory optimization and its potential to enhance overall performance in multi-agent systems, positioning it as a promising approach for UAV-assisted edge computing in dynamic environments. Consequently, this work contributes to advancing decentralized adaptive solutions for multiple unmanned aerial systems in edge computing applications.

In future work, we plan to delve deeper into the effects of energy consumption and communication latency, particularly in terms of how these factors can be integrated and optimized in large-scale scenarios.

## Figures and Tables

**Table 1:** Main notation.

Notation	Definition
U={1,2,…,u}	User equipment set
N={1,2,…,n}	UAV set
τ	Length of the unit time slot
$G_{n}$	Coordinate of the UAV
$G_{u}$	Coordinate of the UE
$d_{u,n,t}$	The distance existing between UE and UAV
$h_{u,n,t}$	Channel gain
$R_{u,n,t}$	Offloading data rate between UE and UAV
$\Gamma_{u,n,t}$	Time taken to offload the task
$f_{n}^{\max}$	Computing resource capacity of the UAV
$f_{u,n,t}$	Resource allocation variable of the UAV
$\beta_{u,n,t}$	Offloading decision
$r_{\max}$	UAV’s maximum coverage

**Table 2:** Values of the parameters.

Definition	Parameter	Value
Noise power	$\sigma^{2}$	−90 dBm
Transmission power	*P*	100 mW
UAV’s maximum speed	$V_{max}$	30 m/s
Task size	*D*	[500, 1000] Kb
Unit channel power gain	μ	5 GHz
UAV’s maximum coverage	$r_{\max}$	50 m
UAV’s computing capacity	$f_{u}^{\max}$	5 GHz
Flight altitude	*Z*	50 m
Bandwidth	*W*	10 MHz

**Table 3:** Comparison of algorithms.

Algorithm	Average Convergence Speed (Steps)	Average Task Success Rate (%)	Notes
QTRAN	$0.54\times 10^{5}$	70.4	Baseline method
Fine-tuned QMIX	$0.22\times 10^{5}$	80.2	Baseline method
K-means QTRAN	$0.25\times 10^{5}$	83.3	Baseline method
LLM-QTRAN	$0.18\times 10^{5}$	93.6	Proposed method

## Data Availability

Data are contained within the article.

## References

[1] Zhao N., Ye Z., Pei Y.-C., Liang Y., Niyato D. (2022). Multi-Agent Deep Reinforcement Learning for Task Offloading in UAV-Assisted Mobile Edge Computing. IEEE Trans. Wirel. Commun..

[2] Ding Y., Feng Y., Lu W., Zheng S., Zhao N., Meng L., Nallanathan A., Yang X. (2023). Online Edge Learning Offloading and Resource Management for UAV-Assisted MEC Secure Communications. IEEE J. Sel. Top. Signal Process..

[3] Liu X., Liu Y., Zhang N., Wu W., Liu A. (2019). Optimizing Trajectory of Unmanned Aerial Vehicles for Efficient Data Acquisition: A Matrix Completion Approach. IEEE Internet Things J..

[4] Haber E.E., Alameddine H.A., Assi C., Sharafeddine S. (2021). UAV-Aided Ultra-Reliable Low-Latency Computation Offloading in Future IoT Networks. IEEE Trans. Commun..

[5] Zhang J., Zhou L., Tang Q., Ngai C.-H., Hu X., Zhao H., Wei J. (2019). Stochastic Computation Offloading and Trajectory Scheduling for UAV-Assisted Mobile Edge Computing. IEEE Internet Things J..

[6] Sunehag P., Lever G., Gruslys A., Czarnecki W.M., Zambaldi V., Jaderberg M., Lanctot M., Sonnerat N., Leibo J.Z., Tuyls K. Value-Decomposition Networks For Cooperative Multi-Agent Learning Based On Team Reward. Proceedings of the 2018 17rd International Conference on Autonomous Agents and Multi-Agent Systems (AAMAS).

[7] Rashid T., Samvelyan M., de Witt C.S., Farquhar G., Foerster J.N., Whiteson S. (2020). Monotonic value function factorisation for deep multi-agent reinforcement learning. J. Mach. Learn. Res..

[8] Son K., Kim D., Kang W.J., Hostallero D.E., Yi Y. QTRAN: Learning to Factorize with Transformation for Cooperative Multi-Agent Reinforcement Learning. https://proceedings.mlr.press/v97/son19a.html.

[9] Wang J., Ren Z., Liu T., Yu Y., Zhang C. (2020). Qplex: Duplex dueling multi-agent q-learning. arXiv.

[10] Pina R., Silva V.D., Hook J., Kondoz A. (2024). Residual Q-Networks for Value Function Factorizing in Multiagent Reinforcement Learning. IEEE Trans. Neural Netw. Learn. Syst..

[11] Ding T., Liu L., Yan Z., Cui L. (2024). Umix: Sustainable Multi-UAV Coordination for Aerial-Terrestrial Networks. IEEE Trans. Netw. Sci. Eng..

[12] Tan S., Chen B., Liu D., Zhang J., Hanzo L. (2023). Communication-Assisted Multi-Agent Reinforcement Learning Improves Task-Offloading in UAV-Aided Edge-Computing Networks. IEEE Wirel. Commun. Lett..

[13] Chen L., Lei Y., Jin S., Zhang Y., Zhang L. (2024). RLingua: Improving Reinforcement Learning Sample Efficiency in Robotic Manipulations With Large Language Models. IEEE Robot. Autom. Lett..

[14] Xu Y., Jian Z., Zha J., Chen X. Poster Abstract: Emergency Networking Using UAVs: A Reinforcement Learning Approach with Large Language Model. Proceedings of the 2024 23rd ACM/IEEE International Conference on Information Processing in Sensor Networks (IPSN).

[15] Han Y., Yang M., Ren Y., Li W. (2024). Large Language Model Guided Reinforcement Learning Based Six-Degree-of-Freedom Flight Control. IEEE Access.

[16] Nascimento N., Alencar P., Cowan D. Self-Adaptive Large Language Model (LLM)-Based Multiagent Systems. Proceedings of the 2023 IEEE International Conference on Autonomic Computing and Self-Organizing Systems Companion (ACSOS-C).

[17] Nascimento N., Alencar P., Cowan D. LLM-MHR: A LLM-Augmented Multimodal Hashtag Recommendation Algorithm. Proceedings of the 2024 IEEE International Conference on Web Services (ICWS).

[18] Wang J., Wang T., Cai W., Xu L., Sun C. (2025). Boosting Efficient Reinforcement Learning for Vision-and-Language Navigation With Open-Sourced LLM. IEEE Robot. Autom. Lett..

[19] Zhang Y., Li J., Chen J., Liu Z., Chen Z. A GCN-Based Decision-Making Network for Autonomous UAV Landing. Proceedings of the 2020 5th International Conference on Advanced Robotics and Mechatronics (ICARM).

[20] Peng H., Mao Z., Jiang B., Cheng Y. (2024). Multiscale Spatial-Temporal Bayesian Graph Conv-Transformer Based Distributed Fault Diagnosis for UAVs Swarm System. IEEE Trans. Aerosp. Electron. Syst..

[21] Wang L., Wang K., Pan C., Xu W., Aslam N., Hanzo L. (2021). Multi-Agent Deep Reinforcement Learning-Based Trajectory Planning for Multi-UAV Assisted Mobile Edge Computing. IEEE Trans. Cogn. Commun. Netw..

[22] Liu Q., Wu J., Xia P., Zhao S., Chen W., Yang Y., Hanzo L. (2016). Charging Unplugged: Will Distributed Laser Charging for Mobile Wireless Power Transfer Work. IEEE Veh. Technol. Mag..

[23] Qu Y., Dai H., Wang H., Dong C., Wu F., Guo S., Wu Q. (2021). Service Provisioning for UAV-Enabled Mobile Edge Computing. IEEE J. Sel. Areas Commun..

[24] Wang L., Wang K., Pan C., Xu W., Aslam N., Nallanathan A. (2022). Deep Reinforcement Learning Based Dynamic Trajectory Control for UAV-Assisted Mobile Edge Computing. IEEE Trans. Mob. Comput..

[25] Zhou F., Wu Y., Hu R.Q., Qian Y. (2018). Computation Rate Maximization in UAV-Enabled Wireless-Powered Mobile-Edge Computing Systems. IEEE J. Sel. Areas Commun..

[26] Hu J., Jiang S., Harding S.A., Wu H., Liao S.W. Rethinking the Implementation Tricks and Monotonicity Constraint in Cooperative Multi-Agent Reinforcement Learning. https://iclr-blogposts.github.io/2023/blog/2023/riit/.

[27] Lu H., Gu C., Luo F., Ding W., Zheng S., Shen Y. (2020). Optimization of Task Offloading Strategy for Mobile Edge Computing Based on Multi-Agent Deep Reinforcement Learning. IEEE Access.

[28] Hu X., Wong K.-K., Yang K., Zheng Z. (2019). UAV-Assisted Relaying and Edge Computing: Scheduling and Trajectory Optimization. IEEE Trans. Wirel. Commun..

[29] Qin Z., Wei Z., Qu Y., Zhou F., Wang H., Ng D.W.K. (2023). AoI-Aware Scheduling for Air-Ground Collaborative Mobile Edge Computing. IEEE Trans. Wirel. Commun..

